# Metabolite profiling reveals a connection between aldehyde dehydrogenase 1A3 and GABA metabolism in breast cancer metastasis

**DOI:** 10.1007/s11306-021-01864-6

**Published:** 2022-01-06

**Authors:** Margaret L. Dahn, Hayley R. Walsh, Cheryl A. Dean, Michael A. Giacomantonio, Wasundara Fernando, J Patrick Murphy, Olivia L. Walker, Marie-Claire D. Wasson, Shashi Gujar, Devanand M. Pinto, Paola Marcato

**Affiliations:** 1 Department of Pathology, Dalhousie University, Halifax, NS Canada; 2 Department of Biology, University of Prince Edward Island, Charlottetown, PEI Canada; 3 Department of Microbiology and Immunology, Dalhousie University, Halifax, NS Canada; 4 Human Health Therapeutics Research Centre, National Research Council of Canada, Halifax, NS Canada; 5 Department of Pathology, Dalhousie University, Rm 11C1, 5850 College Street, Halifax, NS B3H 4R2 Canada

## Abstract

**Introduction:**

Aldehyde dehydrogenase 1A3 (ALDH1A3) is a cancer stem cell (CSC) marker and in breast cancer it is associated with triple-negative/basal-like subtypes and aggressive disease. Studies on the mechanisms of ALDH1A3 in cancer have primarily focused on gene expression changes induced by the enzyme; however, its effects on metabolism have thus far been unstudied and may reveal novel mechanisms of pathogenesis.

**Objective:**

Determine how ALDH1A3 alters the metabolite profile in breast cancer cells and assess potential impacts.

**Method:**

Triple-negative MDA-MB-231 tumors and cells with manipulated ALDH1A3 levels were assessed by HPLC–MS metabolomics and metabolite data was integrated with transcriptome data. Mice harboring MDA-MB-231 tumors with or without altered ALDH1A3 expression were treated with γ-aminobutyric acid (GABA) or placebo. Effects on tumor growth, and lungs and brain metastasis were quantified by staining of fixed thin sections and quantitative PCR. Breast cancer patient datasets from TCGA, METABRIC and GEO were used to assess the co-expression of GABA pathway genes with ALDH1A3.

**Results:**

Integrated metabolomic and transcriptome data identified GABA metabolism as a primary dysregulated pathway in ALDH1A3 expressing breast tumors. Both ALDH1A3 and GABA treatment enhanced metastasis. Patient dataset analyses revealed expression association between ALDH1A3 and GABA pathway genes and corresponding increased risk of metastasis.

**Conclusion:**

This study revealed a novel pathway affected by ALDH1A3, GABA metabolism. Like ALDH1A3 expression, GABA treatment promotes metastasis. Given the clinical use of GABA mimics to relieve chemotherapy-induced peripheral nerve pain, further study of the effects of GABA in breast cancer progression is warranted.

**Supplementary Information:**

The online version contains supplementary material available at 10.1007/s11306-021-01864-6.

## Introduction

Breast cancer survival has improved dramatically with the incorporation of hormone receptor targeted therapies (Brenner et al., 2020). However, there remains a population of patients with aggressive disease who do not benefit from these targeted therapies as their tumors do not express the necessary hormone receptors (estrogen receptor [ER], progesterone receptor [PR], or epidermal growth factor receptor [HER2]). These triple-negative breast cancer (TNBC) patients have an intrinsically more aggressive disease and worse prognosis which is exacerbated by the lack of effective therapies. Although initial response to chemotherapy may be profound, relapse and metastasis occurs frequently and early in TNBC patients (Anders & Carey, 2009). Understanding factors which contribute to the aggressiveness of this subtype and developing therapies that can benefit this population of TNBC patients is essential.

A contributing factor for why TNBC is an aggressive disease is the high aldehyde dehydrogenase 1A3 (ALDH1A3) levels present in the subtype, which promotes tumor progression and metastasis. High ALDH activity (due to ALDH1A3 isoform activity) is a defining feature of breast cancer stem cell (CSC) populations. We and others have shown that high ALDH1A3 expression is associated with worse prognosis, promotes tumor growth, invasion and metastasis, and contributes to chemoresistance in multiple cancers (M.-H. Chen et al., 2016; Cheng et al., 2016; Croker et al., 2017; Duan et al., 2016; Flahaut et al., 2016; Luo et al., 2012; Mao et al., 2013; Marcato et al., 2015; Pérez-Alea et al., 2017; Shao et al., 2014; Thomas et al., 2016; Vidovic et al., 2020; Yang et al., 2013; Zhang et al., 2015). While ALDH1A3 inhibitors are under development, understanding how ALDH1A3 contributes to growth and metastasis could reveal novel avenues for targeting aggressive TNBC and CSCs (Dinavahi et al., 2019).

ALDH1A3 is one of the 19 ALDH isoforms that oxidize aldehydes arising from lipid peroxidation, amino acid catabolism, and xenobiotics (Marchitti et al., 2008). Furthermore, ALDH1A3, 1A1, and 1A2 isoforms generate retinoic acid from vitamin A metabolite retinal. Retinoic acid binds to multiple nuclear hormone receptors leading to expression changes in hundreds of genes, resulting in differentiation, cell cycle arrest or cell proliferation. The transcriptional overlap of ALDH1A3-overexpression and exogenous retinoic acid treatment has been exhaustively documented (Coyle et al., 2017; Flahaut et al., 2016; Luo et al., 2012; Thomas et al., 2016; Vidovic et al., 2020). ALDH1A3’s cancer-promoting activities are least in part related to its generation of retinoic acid and subsequent gene expression changes (Marcato et al., 2015).

Herein, we instead examine the pro-metastasis and cancer progression mechanisms of ALDH1A3 beyond its function in gene expression regulation; the effects of the enzyme on metabolites. Targeted high performance liquid chromatography-mass spectrometry (HPLC–MS) of MDA-MB-231 tumor samples metabolomics revealed a distinct metabolite profile upon ALDH1A3 expression. The differential metabolite and transcriptome data was integrated and revealed a gene expression/metabolite network with γ-aminobutyric acid (GABA) metabolism at the center. ALDH1A3 expression in the breast tumor cells results in decreased GABA and its intermediates N-acetylputrescine and glutamate, suggesting a potential increased utilization of GABA and/or its intermediates by cells expressing high levels of ALDH1A3. Both high ALDH1A3 levels or GABA treatment results in increased metastasis to the lungs and brains of mice bearing orthotopic MDA-MB-231 tumors. In patient tumors, high ALDH1A3 expression is associated with expression of GABA pathway genes. Together the metabolite profiling, in vivo data, and patient tumor analyses, suggests that GABA is pro-metastatic in breast cancer and GABA metabolism and the signaling pathway is connected to ALDH1A3 in breast cancer cells.

## Materials and methods

### Cell culture

Cancer cell lines were obtained from ATCC. MDA-MB-231 cells were grown in Dulbecco’s Modified Eagle Medium (DMEM; Invitrogen) supplemented with 10% Fetal Bovine Serum (FBS; Invitrogen) and antibiotic antimycotic (AA; Invitrogen). ALDH1A3 overexpression (OE) and vector control cells were previously generated and verified (Marcato et al., 2015) and were maintained in media supplemented with 0.25 µg/mL puromycin (Sigma). Cells were cultured in a humidified 37 °C incubator with 5% CO_2_.

### Mass spectrometry-based metabolomics

Metabolites from cultured cells were collected by scraping subconfluent monolayers of cells directly into ice-cold 80% methanol. Metabolites were extracted from tumor samples by crushing 50 mg of a minced tumor into ice-cold 80% methanol. To eliminate large cellular debris, samples were centrifuged at 500×*g* for 5 min and the supernatant was decanted to a new tube. Supernatant was diluted 1/10 with hydrophilic interaction liquid chromatography (HILIC) loading buffer (95% acetonitrile, 2 mM ammonium hydroxide, and 2 mM ammonium acetate). Samples were centrifuged at 13,000×*g* for 5 min to remove any precipitate, and tumor sample supernatants (but not cultured cell sample supernatant) were further diluted 1/10 in HILIC buffer. Next, 50 µL injections were loaded on an Acquity UPLC BEH Amide, 1.7 µm particle size, 2.1 × 100 mm column (Waters #186,004,801). Multiple reaction monitoring (MRM) was performed using a Sciex 5500 QTRAP mass spectrometer using a previously described acquisition method (Yuan et al., 2012). This hybrid dual quadrupole linear ion trap mass spectrometer (MS) has been used previously for quantitative profiling of endogenous polar metabolites in both positive and negative modes from an in vivo source (Yuan et al., 2012). Peak heights for individual Q1/Q3 MRM transitions were extracted using Multiquant Software (Sciex). Peak height data from both ionization modes were normalized independently.

MS total useful signals (MSTUS) (Warrack et al., 2009), a simple MS normalization method, uses total useful metabolite concentrations to normalize metabolite concentrations between samples. Samples were MSTUS normalized on the NOREVA platform (Li et al., 2017). Quantified metabolites are summarized in File S1. The metabolites were normalized and differences in z-scored metabolite levels in different experimental conditions were visualized by hierarchically clustering of the individual replicates by ComplexHeatmap (v2.6.2) R package (Gu et al., 2016). Principal component analysis (PCA) of the metabolite data (File S1) was conducted using the prcomp() function in R v4.0.1 and visualized through the ggbiplot package (v0.55).

### Cell proliferation

ALDH1A3 OE and vector control MDA-MB-231 cells were seeded in 6-well plates and incubated overnight at 37 °C to promote cell adhesion. Adherent MDA-MB-231 cells were stained with 1.25 μM Oregon Green 488 dye (ThermoFisher Scientific) in warm serum-free DMEM for 45 min at 37 °C. To obtain non-proliferative control cells, cells were harvested and fixed in 1% paraformaldehyde. These cells give the highest fluorescence at t = 0 h. The rest of the Oregon Green-stained cells were treated with 50 µM γ-aminobutyric acid (GABA, Sigma-Aldrich) and incubated at 37 °C for 24, 48 and 72 h. At the end of the incubations, the cells were harvested, and the Oregon Green fluorescence was measured using a Celesta instrument (BD Bioscience, Mississauga, ON). The number of cell divisions (n) that took place was calculated using the formula MCF_control_ = 2^n^ × MCF_treatment_. MCF denotes the mean channel fluorescence.

### Mouse studies

All animal experiments were conducted in accordance with the ethical standards and according to the Declaration of Helsinki and according to national and international guidelines and with the Canadian Council on Animal Care standards and a protocol approved by Dalhousie University Committee on Laboratory Animals (#19-013). Eight-week old MDA-MB-231 (2 × 10^6^/mouse), MDA-MB-231 vector control or ALDH1A3-OE (2 × 10^6^/mouse) admixed in 1:1 ratio with phenol red-free high concentration matrigel (BD Bioscience). Resulting tumor growth was quantified (mm^3^; length × width × width/2). Eight- to ten-week-old female NOD/SCID mice, were orthotopically injected with 2 × 10^6^ MDA‐MB‐231 vector control or ALDH1A3-OE cells admixed in 1:1 ratio with phenol red-free high concentration. Mice were intraperitoneally injected daily with 250 µg/kg GABA or the same volume of vehicle (phosphate buffered saline; PBS; Invitrogen).

### Histological analysis of tumors, lungs and brains

The lungs (minus one lobe that was processed for analysis by Reverse Transcriptase Quantitative Polymerase Chain Reaction, RT-QPCR), brains, and tumors were harvested, fixed, paraffin embedded and sectioned (5 µm) for metastasis visualization by haematoxylin and eosin (H&E) staining as described in detail previously (Dahn et al., 2021). Tissue images of three sections per block (1-from the first ¼ of block, 1-from the middle of block, and 1- from the last ¼ of block) were captured on the Zeiss Axio Imager Z1 W/ Color and Monochrome camera at ×2.5 magnification.

To capture the entire lung section, each slide had 4–12 images captured (depending on size of tissue). Images were stitched together using Adobe Photoshop and then imported into the Image J program for metastasis quantification. The total percent metastasis per lung was calculated by averaging percent metastasis of the three quantified thin sections. For the brain sections, incidence of metastasis was scored. Metastatic versus anatomical sites (e.g. islands of Calleja) stained in the thin sections of the mouse brain were confirmed accessing the Brain Explorer 2 software, which is a desktop application for viewing the Allen Mouse Brain Atlas (https://mouse.brain-map.org/static/atlas) (*ALLEN Mouse Brain Atlas, Version 2 (2011)*, 2011).

### Quantification dissemination of metastatic cells to the lungs of mice by human-specific GAPDH RT-QPCR

Following our published method, disseminated metastatic cells were quantified in the left lung lobe by human-specific glyceraldehyde 3-phosphate dehydrogenase (GAPDH) RT-QPCR (Dahn et al., 2021). Briefly, minced left lung lobes were collected in TRIzol and total RNA was purified using a PureLink RNA kit (Thermo Fisher Scientific) following the manufacturer’s instructions. Equal amounts of harvested RNA were reverse transcribed with the iScript cDNA Synthesis Kit (Bio-Rad, Saint Laurent, QC, Canada) as per the manufacturer’s instructions. RT-QPCR was performed using SsoAdvanced Universal SYBR Supermix (Bio-Rad) and human-specific and mouse GAPDH primers (primer sequences are listed in Table S1) as per the manufacturer’s recommended protocol using a CFX384 Touch Real-Time PCR Detection System (Bio-Rad). Primer efficiencies, determined by standard curves of diluted cDNA samples, were incorporated into the CFX Manager software (Bio-Rad). The number of MDA-MB-231 cells detected in the lung lobes was calculated based on a standard curve generated from RNA extracted from naïve lung lobes that had been spiked with increasing numbers of MDA-MB-231 cells (ranging from 10 to 1,000,000 cells).

### Metabolism-focused gene expression node analysis

Microarray gene expression data for MDA-MB-231 cells overexpressing ALDH1A3 versus control cells (n = 3, GSE103426, Affymetrix Human Gene 2.0ST gene array) was incorporated with the metabolite data and network analysis performed using the MetaboAnalyst platform (Pang et al., 2021). 1057 genes (fold change ± 1.2, ANOVA p value < 0.05, File S2) and 71 of 72 metabolites recognized by MetaboAnalyst were inputted into the Network Analysis option to identify gene-metabolite interactions. Gene set enrichment analysis was also performed with the MetaboAnalyst platform.

### Patient dataset analyses

Breast Cancer (METABRIC, Nature 2012 & Nat Commun 2016; n = 1904) and Breast Invasive Carcinoma (TCGA, Cell 2015; n = 816) clinical data and RNA-Seq log2 V2 RSEM gene expression or microarray data were accessed via cBioportal (Cerami et al., 2012; Gao et al., 2013). Microarray-based gene expression of primary breast tumors which had associated clinical data for metastasis was accessed from GSE2034 (n = 286), GSE12276 (n = 204), GSE2603 (n = 81), and GSE5327 (n = 48).

### Statistical analyses

All statistical analyses were performed with GraphPad Prism. In all cases where two experimental conditions are compared, an unpaired student’s t test was performed. When multiple conditions were tested, a two-way ANOVA was performed. Tumor growth (volumes) was modeled using non-linear regression and the slopes of the lines were compared for differences. Pearson's correlation coefficient analysis was conducted on gene expression correlations of patient tumor data. A log-rank test was conducted on Kaplan–Meier survival curves. P values are represented as follows: *=  < 0.05, **=  < 0.01, ***=  < 0.001, ****=  < 0.0001.

## Results

### Metabolomics analysis revealed that GABA degradation is dysregulated by ALDH1A3 expression in MDA-MB-231 tumors

**Fig. 1:**
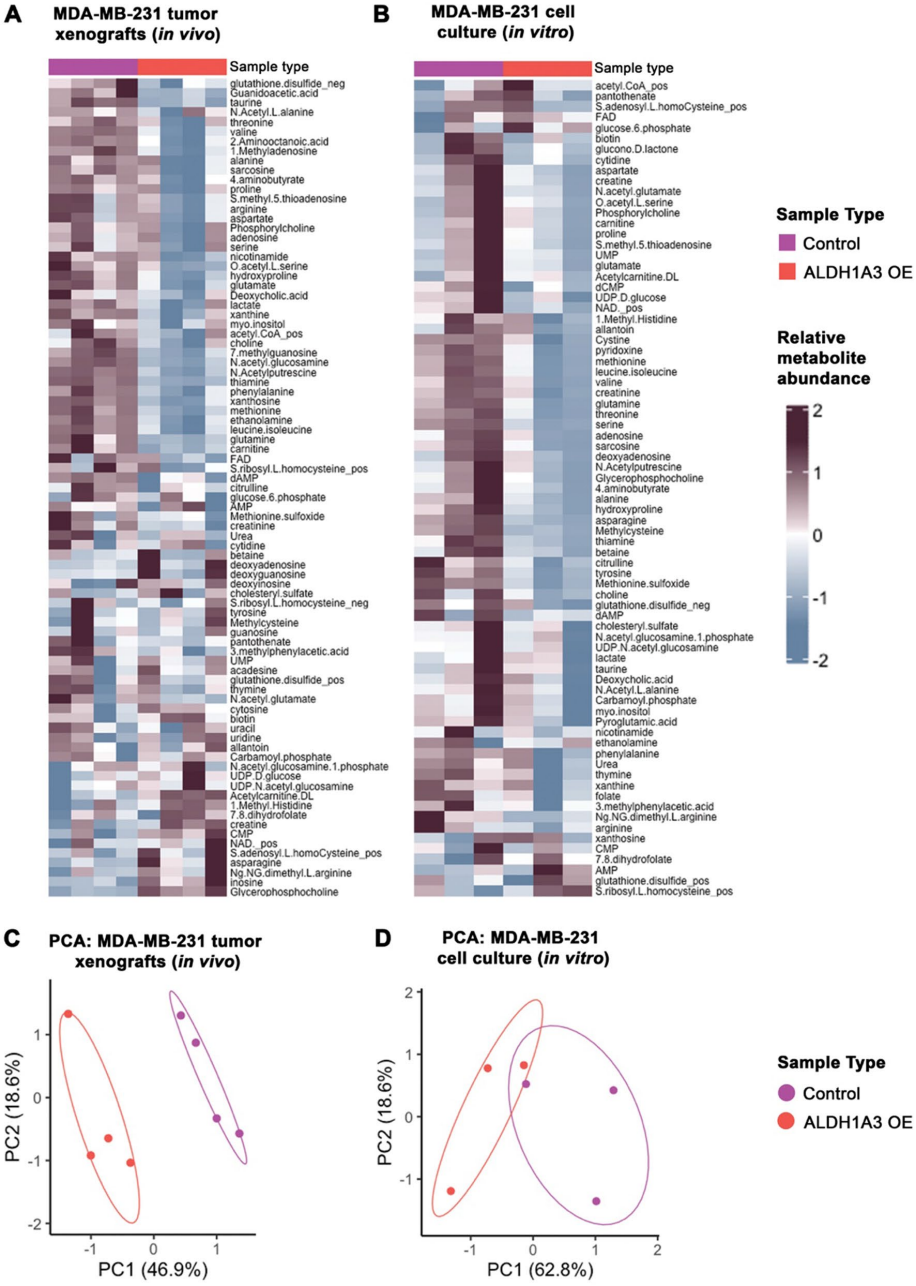
HPLC–MS metabolomics identifies altered metabolic profile in MDA-MB-231 breast tumor cells overexpressing ALDH1A3. **A** and **B** Heatmaps of hierarchically clustered normalized, z-scored metabolite levels detected by metabolomic analysis (File S1). **A** In vivo samples taken from MDA-MB-231 tumors ± ALDH1A3-overexpresion (ALDH1A3-OE) after 53 days of tumor growth (Control n = 4, ALDH1A3-OE n = 4). **B** In vitro samples from MDA-MB-231 cells grown in subconfluent adherent monolayers; n = 3 per group. **C** and **D** Principal component analysis (PCA) of the metabolite in vivo (**C**) and in vitro (**D**) data (File S1)

Complementing the extensive transcriptomics previously completed on ALDH1A3-expressing cancer cells (Coyle et al., 2017; Marcato et al., 2015) with metabolomics is a potential strategy to identify pro-metastatic pathways that are not obvious by gene expression data alone. Of note, MDA-MB-231 cells have limited expression of ALDH1A3, and the overexpression of ALDH1A3 in the TNBC cells has been used to model the effect of high ALDH1A3 levels on the metastasis of TNBCs (Coyle et al., 2017; Marcato et al., 2015; Thomas et al., 2016). HPLC–MS metabolomic analysis of MDA-MB-231 tumors or cells grown in monolayer adherent culture revealed similar metabolite profiles between in vivo and in vitro upon ALDH1A3 overexpression (Fig. 1A, B, File S1). However, the differences in metabolite abundances was magnified in vivo, as the tumor xenografts had a significantly different metabolite profile upon ALDH1A3 overexpression. This was exemplified by the number of differentially abundant metabolites (23/85), and distinguishable subgroups in principal component analysis (PCA) of the tumor samples (Fig. 1C, File S1). Overexpression of ALDH1A3 in the cells grown in culture resulted in a less distinct a metabolite profile, with 4/77 differentially abundant metabolites and incompletely separated subgroups via PCA (Fig. 1D, File S1). This suggested that the impact of ALDH1A3 expression on metabolite levels and metabolism is more impactful the in vivo tumor microenvironment, where nutrients and oxygen are also more limited.

### Gene expression/metabolite interaction network places GABA metabolism at center of ALDH1A3 expression phenotype

**Fig. 2:**
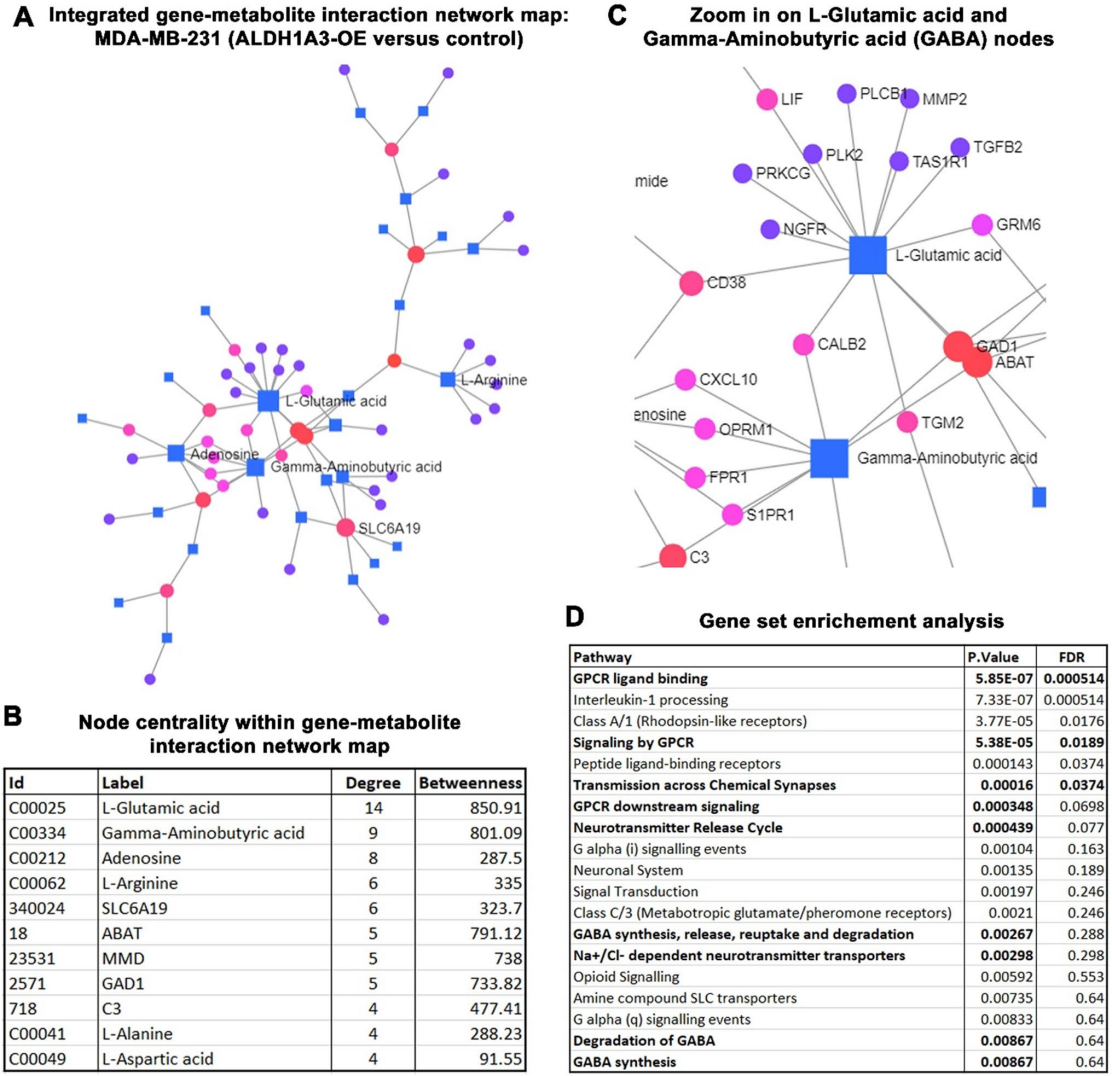
Gene expression/metabolite interaction network places GABA metabolism at center of ALDH1A3 expression phenotype. **A** Gene-metabolite interaction network for MDA-MB-231 ALDH1A3-OE versus control tumors and cells. The network map was generated with the Metaboanalyst software by inputting 1057 genes and associated fold changes and the 71 metabolites and associated fold changes. Metabolites are indicated with squares and genes are indicated with circles. Red circles represent gens that have ≥ 4 node connections, pink circles are genes that have 3–2 node connections and purple circles are genes that have 1 node connection. The size of the square or circle represents the betweenness centrality, where larger symbols have greater betweenness centrality. **B** Node connections and centrality within gene-metabolite interaction map of ALDH1A3-OE tumors/cells (Chong et al., 2018) are summarized for the top nodes. Metabolites have ID numbers starting with C. **C** A zoom in of the top glutamic acid and GABA nodes shows the gene names of the connected nodes. **D** GSEA was performed on the top central subnetwork genes using the MetaboAnalyst software. The top enriched pathways are shown, with pathways related to GABA signaling or metabolism bolded

Given that the metabolome is the product of all gene and translational expression regulation, we explored the link between metabolite changes and altered gene expression that occurs upon ALDH1A3 expression. The altered metabolites in the MDA-MB-231 ALDH1A3 cells/tumors were combined with transcriptome data (identified from 1057 genes with ± 1.2 fold change, p value < 0.05, File S2) to form a transcriptomic-metabolomic map and identify dysregulated pathways (Fig. 2). The central subnetwork contained 71 nodes and the metabolites glutamate (L-glutamic acid) and 4-aminobutyrate (GABA) and genes 4-aminobutyrate aminotransferase (ABAT) and glutamate decarboxylase 1 (GAD1) formed the central nodes (Fig. 2A, B). These components formed a “GABA synthesis/degradation” node and had a high degree centrality (the number of connections made to other nodes) and betweenness centrality (the number of shortest paths going through the node, Fig. 2B, C). Gene set enrichment analysis (GSEA) of the 71 nodes also revealed the most significantly enriched pathways (Fig. 2D; File S3 lists all enriched pathways). The bolded pathways all related to GABA signaling (e.g., the GABA receptors a G-protein coupled receptors (GPCR), and GABA, neurotransmitter, chemical synapses) or GABA synthesis and degradation.

**Fig. 3:**
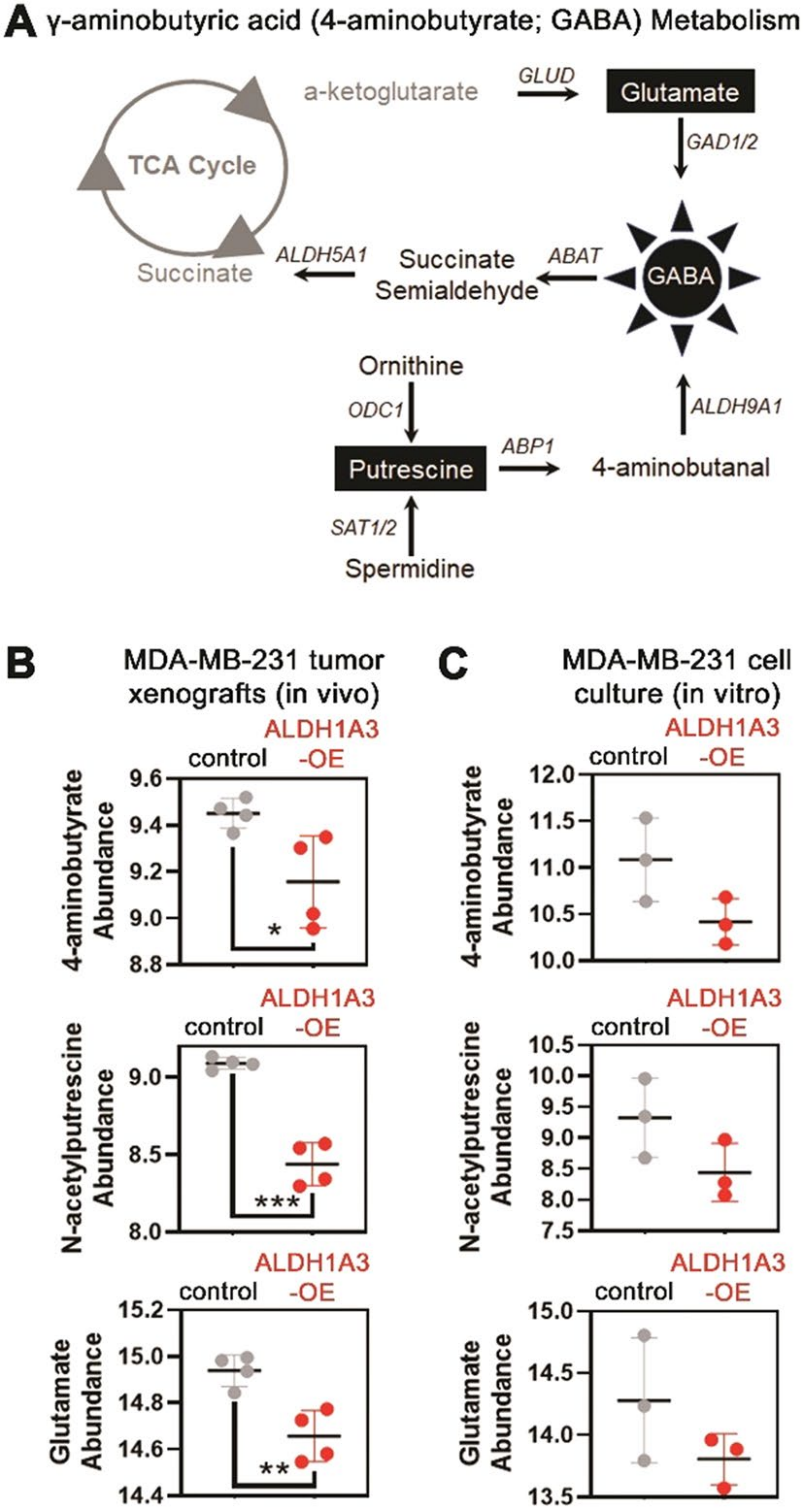
GABA and GABA metabolism intermediates are less abundant in MDA-MB-231 ALDH1A3 overexpression tumors. **A** GABA metabolism with detected metabolites in black boxes. **B**, **C** GABA and GABA intermediate metabolite (N-acetylputrescine and glutamate/L-glutamic acid) abundance in vivo (**B**, n = 4) or in vitro (**C**, n = 3); mean, SD error bars, unpaired two-tailed t-test (p < 0.05*; p < 0.01**, p < 0.001***)

The gene-metabolite network analyses (Fig. 2) placed GABA metabolism (Fig. 3A) as a central component of the ALDH1A3-high phenotype in the breast cancer cells. GABA metabolites (γ-aminobutyric acid and its intermediate metabolites N-acetylputrescine and glutamate) were among the metabolites that were similarly altered in both in vivo (significant) and in vitro (insignificant trend) ALDH1A3-overexpression samples, Fig. 3B). Together this data (Figs. 2 and 3) prioritized the GABA metabolism pathway for further study.

### ALDH1A3 expression and GABA treatment affect tumor growth but not in vitro cell growth

The gene-metabolite interaction network suggested that there is altered GABA signaling/metabolism in ALDH1A3 expressing cells. Metabolomics provides a snapshot of metabolite abundance with little insight into the direction of metabolism. The reduced GABA observed in ALDH1A3 expressing tumors/cells could be due to rapid flux of GABA metabolism based on high usage, or due to decreased GABA metabolism altogether. To determine the impact of GABA on MDA-MB-231 tumors as an oncometabolite/signaling molecule which may affect tumor growth or metastasis, mice harbouring MDA-MB-231 control or ALDH1A3 overexpressing tumors were treated with systemic GABA.

**Fig. 4:**
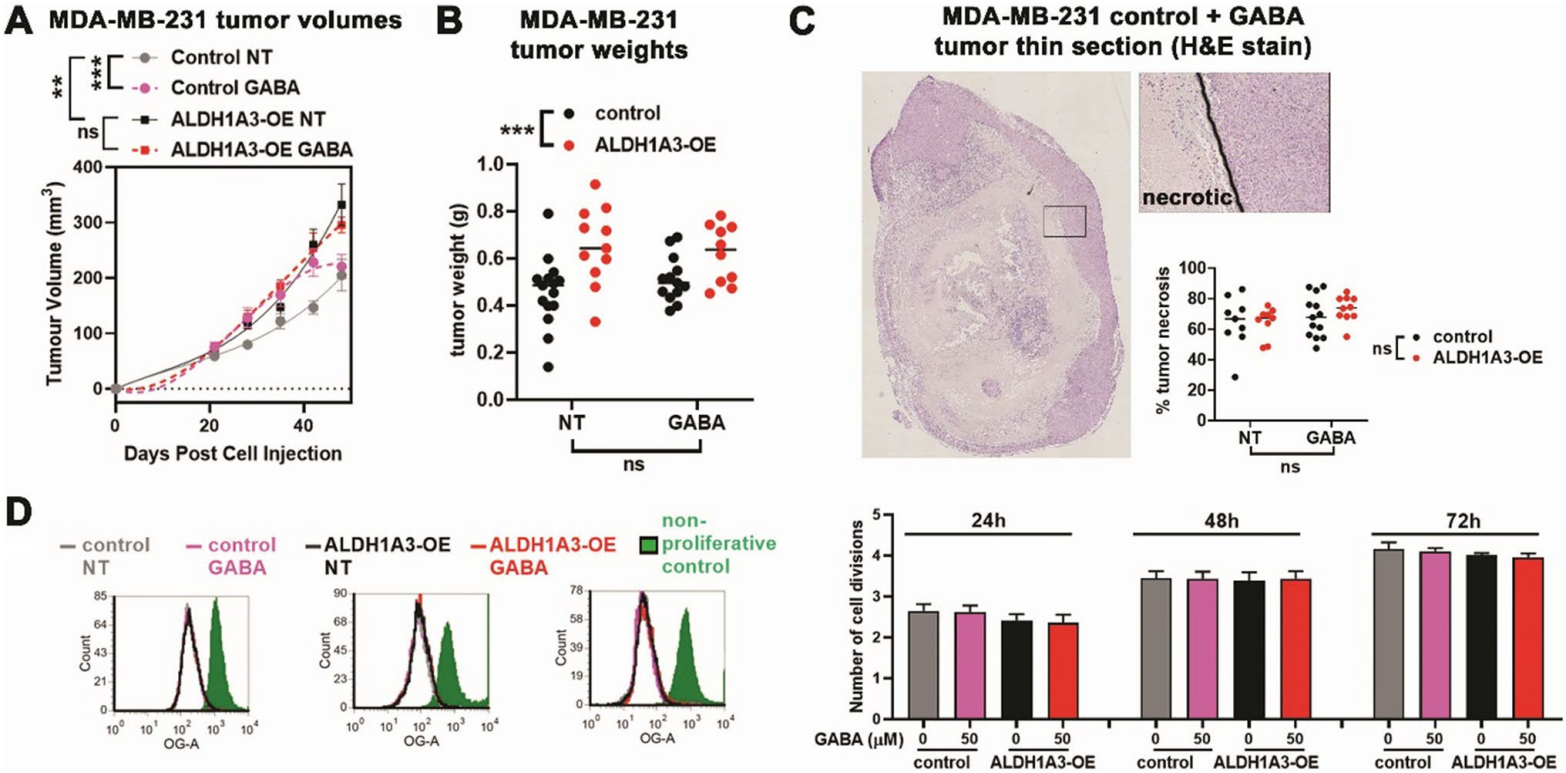
ALDH1A3 overexpression and GABA treatment alter tumor growth dynamics but do not affect cell proliferation rates in vitro. **A** Tumor volumes of orthotopic mammary fat pad implanted MDA-MB-231 cells (control or ALDH1A3 overexpression) and with daily GABA (250 µg/kg) or PBS treatment intraperitoneally in NOD/SCID mice were calculated by caliper measurements (length × width × width/2); Control NT n = 15; Control GABA n = 13; ALDH1A3-OE NT n = 11, ALDH1A3-OE GABA n = 10; SEM error bars). **B** Tumor weights (g) were determined at termination (final day of tumor volumes). **C** % tumor necrosis in H&E-stained thin sections of fixed paraffin embedded was determined by Image J quantification of necrotic areas. Control NT n = 9; Control GABA n = 13; ALDH1A3-OE NT n = 0, ALDH1A3-OE GABA n = 10 (some samples were removed due to poor quality of slides). **D** Cell division over 72 h was monitored by flow cytometry analysis of Oregon Green stained cells, relative to a non-proliferative control (n = 3; SD error bars). Significance in A, B, C and D was determined by two-way ANOVA analysis

As shown previously (Marcato et al., 2015), MDA-MB-231 tumors with ALDH1A3 overexpression resulted in larger tumors in both volume (Fig. 4A) and mass (Fig. 4B) when compared to control tumors. Systemic treatment with 250 µg/kg GABA increased the growth of the tumors early in treatment but this increase growth rate plateaued, which resulted in insignificant tumor weight differences at the end of the experiment (Fig. 4B). Although visible signs of morbidity in some of the mice with ALDH1A3 overexpressing tumors, or treated with GABA, prevented us from extending the experiment to assess the tumor growth dynamics further, we did microscopically assess the harvested tumors. This revealed large zones of necrotic areas, that were not significantly altered by GABA treatment (Fig. 4C). The large proportion of necrosis suggests the tumors were reaching their endpoint in terms of sustainable kinetic growth. Likely the ALDH1A3 overexpression provides an additional advantage not imparted by GABA treatment that better supports the increased growth of the tumor cells.

We also assessed the effects of ALDH1A3 overexpression or GABA treatment in cell culture. Notably, neither ALDH1A3 expression nor GABA treatment affected cell proliferation in vitro (Fig. 4D), suggesting that ALDH1A3/GABA metabolism effects in the TNBC model are at least in part dependent upon the tumor microenvironment. This is consistent with our metabolite analysis where differences elicited by ALDH1A3 in overall metabolite changes and GABA metabolism are not significant under in vitro cell culturing conditions (Figs. 1 and 3).

### ALDH1A3 expression and GABA treatment enhance metastasis

**Fig. 5:**
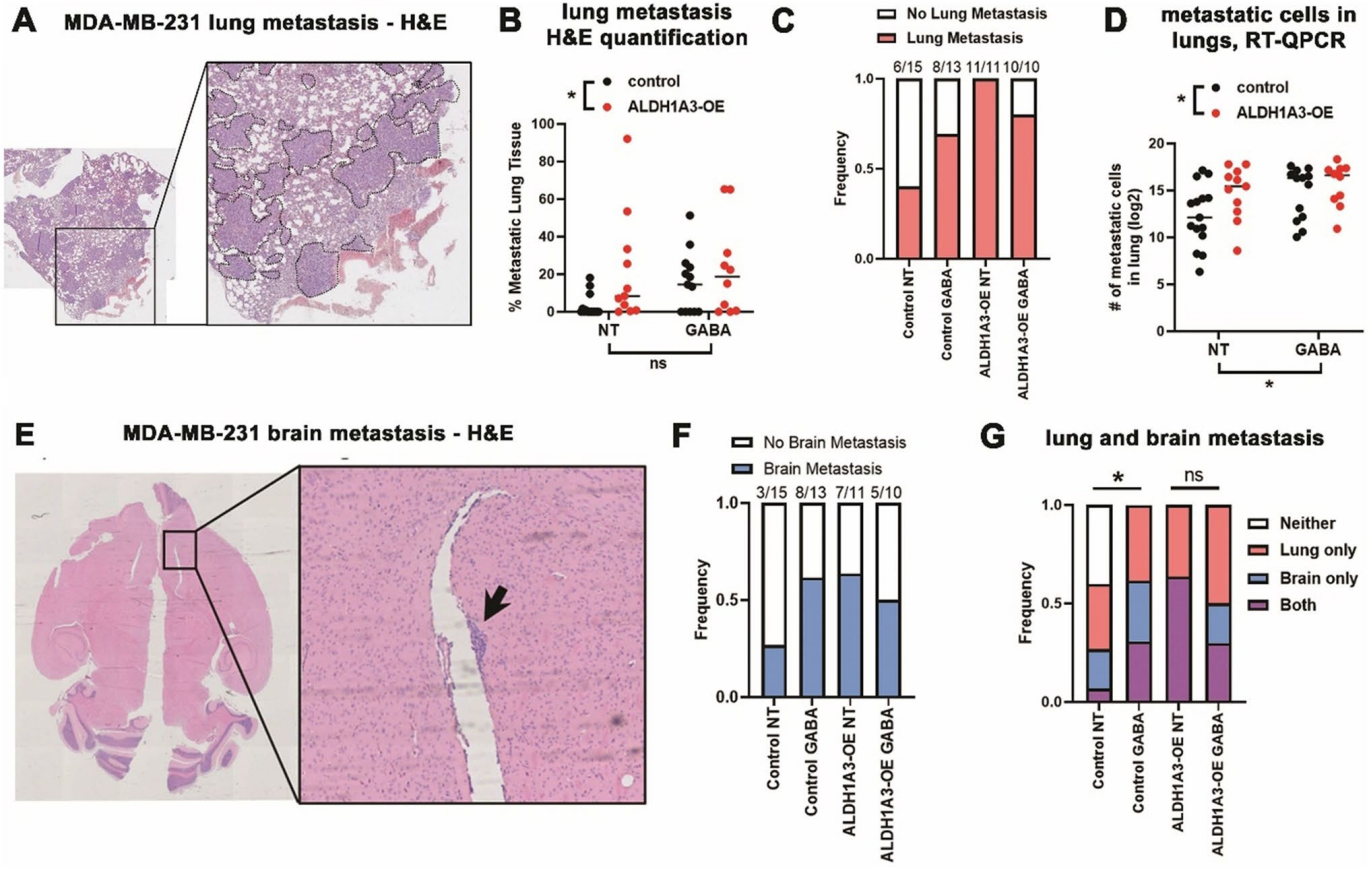
ALDH1A3 expression and GABA treatment increase metastasis of orthotopic MDA-MB-231 primary tumors. **A** Light microscopy image of H&E-stained lung section; example from GABA-treated control tumor bearing mouse with metastatic areas outlined. **B** Histology quantified lung metastasis (bar = median); two-way ANOVA p < 0.05*. **C** Incidence of metastasis was determined calculating the number of lungs in each group that had any detectable presence of metastasis by histological examination. **D** Human-specific GAPDH RT-QPCR quantified disseminated metastatic cells to the lungs of the mice (bar = median, log2 values plotted); two-way ANOVA p < 0.05*.** E** Light microscopy image of an H&E-stained brain section; example from GABA-treated MDA-MB-231 tumor-bearing mouse with metastatic area indicated by the arrow. **F** Incidence of metastasis was determined by calculating the number of brains in each group that had any detectable presence of metastasis by histological examination. **G** Incidence of any metastasis determined by calculating the number of mice in each group that had any detectable presence of metastasis in the lungs, brain or both the brain and the lungs by histological examination (Fisher’s exact test, p < 0.05*, ns = non-significant)

We assessed the impact of ALDH1A3 expression and/or GABA treatment metastasis by histological H&E staining of fixed lung sections (Fig. 5A). Quantification of the stained fixed lung sections demonstrated increased metastasis upon ALDH1A3 overexpression (Fig. 5B) Though the mean percentage of metastatic lung tissue was not significantly different between untreated and GABA-treated mice harbouring control tumors, both GABA and ALDH1A3 increased the number of lung tissue in which we detected metastasis by H&E staining of fixed lung sections (6/15 versus 8/13 for untreated versus GABA treated, Fig. 5C). The incidence of metastasis was similar in GABA-treated mice (8/10; 80%) versus untreated mice (11/11; 100%) harbouring ALDH1A3 overexpressing tumors (Fig. 5C).

We recently reported an RT-QPCR-based method which is more sensitive than histological analysis for detecting distal metastatic cells in xenograft tumor murine models (Dahn et al., 2021). The method can detect 10 disseminated MDA-MB-231 cells in a lung lobe. Utilizing this more sensitive method, we detected significantly increased metastatic cells in the lungs of mice bearing MDA-MB-231 tumors with ALDH1A3 overexpression or treated with systemic GABA (Fig. 5D).

Next, we assessed the effects of ALDH1A3 and GABA treatment on metastasis to the brain. Mouse brains were formalin fixed and paraffin embedded, and sections stained by H&E and imaged (Fig. 5E). GABA treatment and ALDH1A3 overexpression similarly increased the number of mice with detectable brain metastasis by histological examination (Fig. 5F). Overall incidence of metastasis was increased upon GABA treatment and reached similar levels as ALDH1A3 overexpression (Fig. 5G).

### Expression of GABA metabolizing enzyme ABAT is lower in patient breast tumors with high ALDH1A3 expression and in patient breast tumors that metastasize

**Fig. 6:**
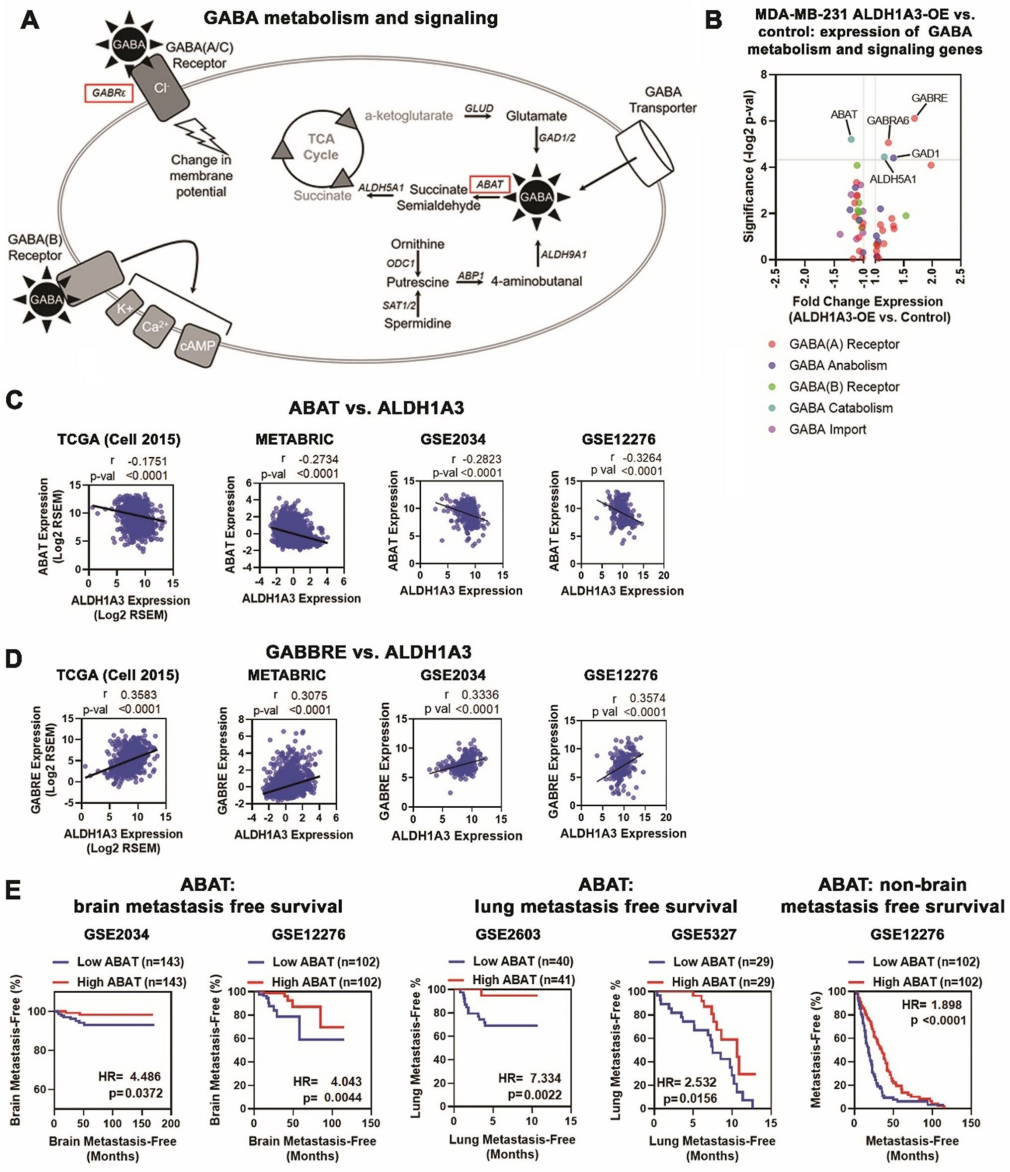
GABA signaling/metabolism gene expression associated with ALDH1A3 expression in MDA-MB-231 cells and in patient breast tumors and associations with metastasis. **A** The GABA signaling/metabolism pathway with receptors, channels, and metabolism. **B** Transcriptome analysis of MDA-MB-231 cells (ALDH1A3 overexpression versus control cells) completed by Affymetrix Human Gene 2.0ST Array (n = 3) identifed differential expression of some GABA signaling/metabolism; grey line indicates p < 0.05.** C** and **D** Expression of GABA signaling pathway players (ABAT, **C** and and GABRE, **D**) significantly correlated with ALDH1A3 in breast cancer patient tumors. The datasets analyzed are METABRIC (n = 1904); TCGA, Cell 2015 (n = 816; RNA-Seq RSEM log2); GSE2034 (Affymetrix Human Genome U133A Array log2 expression from primary tumors of 286 lymph node negative breast cancer patients); GSE12276 (Affymetrix Human Genome U133 Plus 2.0 Array log2 expression of 204 primary breast tumors from patients all of whom experienced local or distant relapse); r values are based on Pearson correlations. **E** High and low expression of ABAT was categorized based on median expression for Kaplan–Meier metastasis-free survival analysis to the brain, lungs or to sites other than the brain for GSE2034, GSE12276, GSE2603 (Affymetrix Human Genome U133A Array log2 expression from primary tumors of 81 breast cancer patients), and GSE5327 (Affymetrix Human Genome U133A Array log2 expression from 48 primary ER- breast tumors). HR = hazard ratio, p value calculated by log-rank test

The results thus far indicated that GABA treatment can increase metastasis of MDA-MB-231 tumors to levels induced by ALDH1A3 overexpression (Fig. 4). To further delineate the potential interaction between GABA signaling/metabolism and ALDH1A3 in breast cancer we assessed the transcriptome data for MDA-MB-231 cells and patient tumors for consistent differential expression of GABA signaling and metabolism genes (Fig. 6A) associated with high ALDH1A3 expression.

In MDA-MB-231 ALDH1A3 overexpressing cells there was significantly reduced expression of γ-aminobutyrate aminotransferase (ABAT); with significant up-regulation of γ-aminobutyric acid receptor subunit ε (GABRE), γ-aminobutyric acid receptor subunit α-6 (GABRA6), GAD1, and succinic semialdehyde dehydrogenase (ALDH5A1, Fig. 6B).

In breast cancer patient datasets (TCGA, Cell 2015 and METABRIC), expression of ABAT was negatively correlated (Fig. 6C) while expression of GABRE was positively correlated (Fig. D) with ALDH1A3 expression, consistent with the MDA-MB-231 data (Fig. 6B). Neither GABRA6, GAD1, or ALDH5A1 were significantly and consistently correlated with ALDH1A3 expression in the TCGA or METABRIC datasets (Pearson r: GABRA6 − 0.0528 & 0.0117; GAD1 − 0.0343 & − 0.1308; ALDH5A1 0.0794 & − 0.2195 respectively). We further confirmed the association of ABAT or GABRE expression with high ALDH1A3 in two independent datasets of primary breast tumors that later developed distal metastasis (GSE2034 and GSE12276). Expression of ABAT was again negatively correlated (Fig. 6C) while GABRE was positively correlated (Fig. 6D) with ALDH1A3 expression. In both patient groups, low ABAT expression in the primary breast tumor was significantly and strongly associated with increased risk of brain metastasis (Fig. 6E), while GABRE was not (Fig. S1). Furthermore, when primary breast tumors had low ABAT expression, there was a higher risk for lung-specific metastasis and all distal sites excluding brain (Fig. 6E), but GABRE expression did not impact the risk of distant metastasis (Fig. S1). Together, this brain and lung metastasis data suggested that ABAT-low primary breast tumors (often with concurrent high ALDH1A3 expression) are highly metastatic and are agnostic to whether the metastatic site is enriched for GABA (i.e., the brain) or is depleted for GABA (i.e., the lung).

## Discussion

In this study, the effect of ALDH1A3 on the metabolome of TNBC was assessed in conjunction with transcriptome data analysis. This was done to identify novel ways in which ALDH1A3 contributes to the aggressive nature of TNBC cells. The significant associations in terms of metabolite changes in only the context of the in vivo tumor samples in comparison to the in vitro samples, suggested that the comparably nutrient poor tumor microenvironment is an important factor when assessing the impact of ALDH1A3 on the metabolome. The mass spectroscopy-based metabolomics on TNBC MDA-MB-231 tumors and cells, with or without ALDH1A3 overexpression, revealed ALDH1A3-dependent changes in the metabolism of the inhibitory neurotransmitter γ-aminobutyric acid (GABA).

In our studies, assessing the potential of ALDH1A3 and GABA on the TNBC, the most obvious effects was the impact of both ALDH1A3 and GABA in the increased metastasis to the lungs and brains of tumor-bearing animals. In terms of effects of tumor growth and cell proliferation, effects of ALDH1A3/GABA on MDA-MB-231 growth were restricted to in vivo conditions, where ALDH1A3 expression increased tumor growth overall, and GABA appeared to increase tumor growth initially, although this effect was lost at the end of the experiment. The high levels of tumor necrosis in the harvested tumors suggests that as the tumors grew to a larger size, they lacked sufficient supports in the tumor microenvironment to ensure sustained proliferation. In tumors overexpressing ALDH1A3, the increase in tumor growth was sustained until the end of the experiments, while GABA treatment was insufficient to sustain the increased tumor growth. This suggests that ALDH1A3 has effects in the tumor microenvironment that are not fully explained by ALDH1A3’s effects on GABA metabolism and signaling.

Interestingly, we did not find that ALDH1A3 expression, nor GABA treatment, increased growth in vitro, suggesting that additional factors present (or absent) in the tumor microenvironment are required for mediating pro-growth effects of ALDH1A3 or GABA in MDA-MB-231 cells. This is distinct from the findings of some, who have reported that GABA treatment, GABA metabolism and GABAergic signaling does increase the in vitro proliferation of cancer cells (Hujber et al., 2018; Kanbara et al., 2018; Neman et al., 2014; Takehara et al., 2007; Young & Bordey, 2009). Based on the finds of others and our study, we can conclude that both cellular context and tumor microenvironment are important determinants in the pro-tumorigenic effects of GABA metabolism and GABAergic signaling.

In the context of GABA signaling and metabolism, the transcriptome analysis of MDA-MB-231 cells revealed ALDH1A3-dependent changes in expression of some GABA signaling components, including upregulation of receptor subunits (GABRE) and downregulation of GABA catabolic enzyme ABAT. These gene expression changes were reflected in breast cancer patient tumors. The importance of GABA signaling/metabolism in breast cancer metastasis is illustrated by the four independent patient datasets wherein decreased ABAT expression (and inferred increased GABA signaling/metabolism) was strongly and significantly associated with an increased risk of brain and lung metastasis.

As a major inhibitory neurotransmitter in the central nervous system, GABA is most abundant in the brain (Bowery & Smart, 2006). Aside from its main role as an inhibitory neurotransmitter, GABA has been shown to moonlight as a pro-growth and pro-metastasis molecule. In TNBC cells with aberrant GABA metabolism, there is an enhanced ability to metastasize to the brain (Neman et al., 2014). The GABAergic signaling pathway plays an important role in metastasis of breast cancer, especially to the brain (Brzozowska et al., 2017; Chen et al., 2019; Gumireddy et al., 2016; Neman et al., 2014; Sizemore et al., 2014). Importantly, here we found that systemic GABA treatment and ALDH1A3 overexpression increased lung and brain metastasis of orthotopic MDA-MB-231 tumors.

Though it is considered an aggressive and metastatic TNBC model, MDA-MB-231 cells typically do not readily metastasize to the brains of mice. Indeed, MDA-MB-231 brain metastasis models follow two paradigms: (1) MDA-MB-231 cells are injected intracardially or (2) intracranially (El-Mabhouh et al., 2008; Gong et al., 2019; Kijewska et al., 2019; Sayyad et al., 2019). Even the “brain-seeking” MDA-MB-231BR cell line—derived from intracardiac-injected MDA-MB-231 cells which formed a brain lesion—must be injected intracranially to reliably form brain metastases (Delaney et al., 2019; Masiero et al., 2019; Yoneda et al., 2001). The increased systemic GABA or ALDH1A3 levels increased the permissiveness of the breast cancer cells to metastasize to the brain.

If ALDH1A3 and/or increased GABA signaling/metabolism primes TNBC cells to establish brain or lung metastases, some common clinical practices may need to be re-assessed. Peripheral nerve pain is a dose-limiting and long-lasting side effect observed in 31–42% of patients receiving taxane chemotherapy (Ghoreishi et al., 2018; Mustafa Ali et al., 2017). These individuals are eligible to receive treatment with gabapentin which is a structural analog of GABA and is able to dampen neuropathic pain (Aghili et al., 2019; Arai et al., 2010; Ross et al., 2005; Tsavaris et al., 2008; Xiao et al., 2007). Though the complete mechanism of action is still under investigation, gabapentin does not act through GABA(A) or GABA(B) receptor binding, but by indirectly increasing intracellular GABA synthesis (Sills, 2006). Further investigation of the ALDH1A3/GABA axis is needed to determine whether patients may be at increased risk of metastasis if given gabapentin or other GABA agonists.

## Supplementary Information

Below is the link to the electronic supplementary material.

Supplementary file1 (XLSX 45 kb)

Supplementary file2 (XLSX 42 kb)

Supplementary file3 (CSV 3 kb)

Supplementary file4 (PDF 125 kb)

## Declarations

### Research involving animals

All procedures performed in studies involving animals were in accordance with ethical standards and according to the Declaration of Helsinki and according to national and international guidelines and with the Canadian Council on Animal Care standards and a protocol approved by Dalhousie University Committee on Laboratory Animals (#19-013).

## References

[CR1] Aghili M, Zare M, Mousavi N, Ghalehtaki R, Sotoudeh S, Kalaghchi B, Akrami S, Esmati E (2019). Efficacy of gabapentin for the prevention of paclitaxel induced peripheral neuropathy: A randomized placebo controlled clinical trial. The Breast Journal.

[CR2] *ALLEN Mouse Brain Atlas, version 2 (2011)* (pp. 1–72). (2011). The Allen Institute for Brain Science. https://mouse.brain-map.org/static/atlas

[CR3] Anders CK, Carey LA (2009). Biology, metastatic patterns, and treatment of patients with triple-negative breast cancer. Clinical Breast Cancer.

[CR4] Arai YCP, Matsubara T, Shimo K, Suetomi K, Nishihara M, Ushida T, Kobayashi K, Suzuki C, Kinoshita A, Kondo M, Matsubara S, Hayashi R, Tohyama Y, Nishida K, Arakawa M (2010). Low-dose gabapentin as useful adjuvant to opioids for neuropathic cancer pain when combined with low-dose imipramine. Journal of Anesthesia.

[CR5] Bowery NG, Smart TG (2006). GABA and glycine as neurotransmitters: A brief history. British Journal of Pharmacology.

[CR6] Brenner DR, Weir HK, Demers AA, Ellison LF, Louzado C, Shaw A, Turner D, Woods RR, Smith LM (2020). Projected estimates of cancer in Canada in 2020. CMAJ.

[CR7] Brzozowska A, Burdan F, Duma D, Solski J, Mazurkiewicz M (2017). γ-Amino butyric acid (GABA) level as an overall survival risk factor in breast cancer. Annals of Agricultural and Environmental Medicine.

[CR8] Cerami E, Gao J, Dogrusoz U, Gross BE, Sumer SO, Aksoy BA, Jacobsen A, Byrne CJ, Heuer ML, Larsson E, Antipin Y, Reva B, Goldberg AP, Sander C, Schultz N (2012). The cBio cancer genomics portal: An open platform for exploring multidimensional cancer genomics data. Cancer Discovery.

[CR9] Chen M-H, Weng J-J, Cheng C-T, Wu R-C, Huang S-C, Wu C-E, Chung Y-H, Liu C-Y, Chang M-H, Chen M-H, Chiang K-C, Yeh T-S, Su Y, Yeh C-N (2016). ALDH1A3, the major aldehyde dehydrogenase isoform in human cholangiocarcinoma cells, affects prognosis and gemcitabine resistance in cholangiocarcinoma patients. Clinical Cancer Research.

[CR10] Chen X, Cao Q, Liao R, Wu X, Xun S, Huang J, Dong C (2019). Loss of ABAT-mediated GABAergic system promotes basal-like breast cancer progression by activating Ca2+-NFAT1 axis. Theranostics.

[CR11] Cheng P, Wang J, Waghmare I, Sartini S, Coviello V, Zhang Z, Kim SH, Mohyeldin A, Pavlyukov MS, Minata M, Valentim CLL, Chhipa RR, Bhat KPL, Dasgupta B, La Motta C, Kango-Singh M, Nakano I (2016). FOXD1-ALDH1A3 signaling is a determinant for the self-renewal and tumorigenicity of mesenchymal glioma stem cells. Cancer Research.

[CR12] Chong J, Soufan O, Li C, Caraus I, Li S, Bourque G, Wishart DS, Xia J (2018). MetaboAnalyst 4.0: towards more transparent and integrative metabolomics analysis. Nucleic Acids Research.

[CR13] Coyle KM, Maxwell S, Thomas ML, Marcato P (2017). Profiling of the transcriptional response to all-Trans retinoic acid in breast cancer cells reveals RARE-independent mechanisms of gene expression. Scientific Reports.

[CR14] Croker AK, Rodriguez-Torres M, Xia Y, Pardhan S, Leong HS, Lewis JD, Allan AL (2017). Differential functional roles of ALDH1A1 and ALDH1A3 in mediating metastatic behavior and therapy resistance of human breast cancer cells. International Journal of Molecular Sciences.

[CR15] Dahn ML, Dean CA, Jo DB, Coyle KM, Marcato P (2021). Human-specific GAPDH qRT-PCR is an accurate and sensitive method of xenograft metastasis quantification. Molecular Therapy - Methods and Clinical Development.

[CR16] Delaney LJ, Ciraku L, Oeffinger BE, Wessner CE, Liu JB, Li J, Nam K, Forsberg F, Leeper DB, O’Kane P, Wheatley MA, Reginato MJ, Eisenbrey JR (2019). Breast cancer brain metastasis response to radiation after microbubble oxygen delivery in a murine model. Journal of Ultrasound in Medicine : Official Journal of the American Institute of Ultrasound in Medicine.

[CR17] Dinavahi, S. S., Bazewicz, C. G., Gowda, R., & Robertson, G. P. (2019). Aldehyde Dehydrogenase Inhibitors for Cancer Therapeutics. In *Trends in pharmacological sciences* (Vol. 40, Issue 10, pp. 774–789). Elsevier Ltd. 10.1016/j.tips.2019.08.002 10.1016/j.tips.2019.08.002 31515079

[CR18] Duan J-J, Cai J, Guo Y-F, Bian X-W, Yu S-C (2016). ALDH1A3, a metabolic target for cancer diagnosis and therapy. International Journal of Cancer.

[CR19] El-Mabhouh AA, Nation PN, Kaddoura A, Mercer JR (2008). Unexpected preferential brain metastases with a human breast tumor cell line MDA-MB-231 in BALB/c nude mice. Veterinary Pathology.

[CR20] Flahaut M, Jauquier N, Chevalier N, Nardou K, Balmas Bourloud K, Joseph J-M, Barras D, Widmann C, Gross N, Renella R, Mühlethaler-Mottet A (2016). Aldehyde dehydrogenase activity plays a Key role in the aggressive phenotype of neuroblastoma. BMC Cancer.

[CR21] Gao J, Aksoy BA, Dogrusoz U, Dresdner G, Gross B, Sumer SO, Sun Y, Jacobsen A, Sinha R, Larsson E, Cerami E, Sander C, Schultz N (2013). Integrative analysis of complex cancer genomics and clinical profiles using the cBioPortal. Science Signaling.

[CR22] Ghoreishi Z, Keshavarz S, Asghari Jafarabadi M, Fathifar Z, Goodman KA, Esfahani A (2018). Risk factors for paclitaxel-induced peripheral neuropathy in patients with breast cancer 11 Medical and Health Sciences 1112 Oncology and Carcinogenesis. BMC Cancer.

[CR23] Gong X, Hou Z, Endsley MP, Gronseth EI, Rarick KR, Jorns JM, Yang Q, Du Z, Yan K, Bordas ML, Gershan J, Deepak P, Geethadevi A, Chaluvally-Raghavan P, Fan Y, Harder DR, Ramchandran R, Wang L (2019). Interaction of tumor cells and astrocytes promotes breast cancer brain metastases through TGF-β2/ANGPTL4 axes. Npj Precision Oncology.

[CR24] Gu Z, Eils R, Schlesner M (2016). Complex heatmaps reveal patterns and correlations in multidimensional genomic data. Bioinformatics.

[CR25] Gumireddy K, Li A, Kossenkov AV, Sakurai M, Yan J, Li Y, Xu H, Wang J, Zhang PJ, Zhang L, Showe LC, Nishikura K, Huang Q (2016). The mRNA-edited form of GABRA3 suppresses GABRA3-mediated Akt activation and breast cancer metastasis. Nature Communications.

[CR26] Hujber Z, Horváth G, Petővári G, Krencz I, Dankó T, Mészáros K, Rajnai H, Szoboszlai N, Leenders WPJ, Jeney A, Tretter L, Sebestyén A (2018). GABA, glutamine, glutamate oxidation and succinic semialdehyde dehydrogenase expression in human gliomas. Journal of Experimental & Clinical Cancer Research.

[CR27] Kanbara K, Otsuki Y, Watanabe M, Yokoe S, Mori Y, Asahi M, Neo M (2018). GABAB receptor regulates proliferation in the high-grade chondrosarcoma cell line OUMS-27 via apoptotic pathways. BMC Cancer.

[CR28] Kijewska M, Viski C, Turrell F, Fitzpatrick A, Van Weverwijk A, Gao Q, Iravani M, Isacke CM (2019). Using an in-vivo syngeneic spontaneous metastasis model identifies ID2 as a promoter of breast cancer colonisation in the brain. Breast Cancer Research.

[CR29] Li B, Tang J, Yang Q, Li S, Cui X, Li Y, Chen Y, Xue W, Li X, Zhu F (2017). NOREVA: Normalization and evaluation of MS-based metabolomics data. Nucleic Acids Research.

[CR30] Luo Y, Dallaglio K, Chen Y, Robinson WA, Robinson SE, McCarter MD, Wang J, Gonzalez R, Thompson DC, Norris DA, Roop DR, Vasiliou V, Fujita M (2012). ALDH1A isozymes are markers of human melanoma stem cells and potential therapeutic targets. Stem Cells.

[CR31] Mao P, Joshi K, Li J, Kim SH, Li P, Santana-Santos L, Luthra S, Chandran UR, Benos PV, Smith L, Wang M, Hu B, Cheng SY, Sobol RW, Nakano I (2013). Mesenchymal glioma stem cells are maintained by activated glycolytic metabolism involving aldehyde dehydrogenase 1A3. Proceedings of the National Academy of Sciences.

[CR32] Marcato P, Dean CA, Liu R-Z, Coyle KM, Bydoun M, Wallace M, Clements D, Turner C, Mathenge EG, Gujar SA, Giacomantonio CA, Mackey JR, Godbout R, Lee PWK (2015). Aldehyde dehydrogenase 1A3 influences breast cancer progression via differential retinoic acid signaling. Molecular Oncology.

[CR33] Marchitti SA, Brocker C, Stagos D, Vasiliou V (2008). Non-P450 aldehyde oxidizing enzymes: The aldehyde dehydrogenase superfamily. Expert Opinion on Drug Metabolism & Toxicology.

[CR34] Masiero M, Li D, Whiteman P, Bentley C, Greig J, Hassanali T, Watts S, Stribbling S, Yates J, Bealing E, Li JL, Chillakuri C, Sheppard D, Serres S, Sarmiento-Soto M, Larkin J, Sibson NR, Handford PA, Harris AL, Banham AH (2019). Development of therapeutic anti-Jagged1 antibodies for cancer therapy. Molecular Cancer Therapeutics.

[CR35] Mustafa Ali M, Moeller M, Rybicki L, Moore HCF (2017). Long-term peripheral neuropathy symptoms in breast cancer survivors. Breast Cancer Research and Treatment.

[CR36] Neman J, Termini J, Wilczynski S, Vaidehi N, Choy C, Kowolik CM, Li H, Hambrecht AC, Roberts E, Jandial R (2014). Human breast cancer metastases to the brain display GABAergic properties in the neural niche. Proceedings of the National Academy of Sciences of the United States of America.

[CR37] Pang Z, Chong J, Zhou G, de Lima Morais DA, Chang L, Barrette M, Gauthier C, Jacques P-É, Li S, Xia J (2021). MetaboAnalyst 5.0: narrowing the gap between raw spectra and functional insights. Nucleic Acids Research.

[CR38] Pérez-Alea M, Mcgrail K, Sánchez-Redondo S, Ferrer B, Fournet G, Cortés J, Muñoz E, Hernandez-Losa J, Tenbaum S, Martin G, Costello R, Ceylan I, Garcia-Patos V, Recio JA (2017). ALDH1A3 is epigenetically regulated during melanocyte transformation and is a target for melanoma treatment. Oncogene.

[CR39] Ross JR, Goller K, Hardy J, Riley J, Broadley K, A’Hern R, Williams J (2005). Gabapentin is effective in the treatment of cancer-related neuropathic pain: A prospective, open-label study. Journal of Palliative Medicine.

[CR40] Sayyad MR, Puchalapalli M, Vergara NG, Wangensteen SM, Moore M, Mu L, Edwards C, Anderson A, Kall S, Sullivan M, Dozmorov M, Singh J, Idowu MO, Koblinski JE (2019). Syndecan-1 facilitates breast cancer metastasis to the brain. Breast Cancer Research and Treatment.

[CR41] Shao C, Sullivan JP, Girard L, Augustyn A, Yenerall P, Rodriguez-Canales J, Liu H, Behrens C, Shay JW, Wistuba II, Minna JD (2014). Essential role of aldehyde dehydrogenase 1A3 for the maintenance of non-small cell lung cancer stem cells is associated with the STAT3 pathway. Clinical Cancer Research.

[CR42] Sills, G. J. (2006). The mechanisms of action of gabapentin and pregabalin. In *Current opinion in pharmacology* (Vol. 6, Issue 1 SPEC. ISS., pp. 108–113). Elsevier Ltd. 10.1016/j.coph.2005.11.003 10.1016/j.coph.2005.11.003 16376147

[CR43] Sizemore GM, Sizemore ST, Seachrist DD, Keri RA (2014). GABA(A) receptor Pi (GABRP) stimulates basal-like breast cancer cell migration through activation of extracellular-regulated kinase 1/2 (ERK1/2). Journal of Biological Chemistry.

[CR44] Takehara A, Hosokawa M, Eguchi H, Ohigashi H, Ishikawa O, Nakamura Y, Nakagawa H (2007). γ-aminobutyric acid (GABA) stimulates pancreatic cancer growth through overexpressing GABAA receptor π subunit. Cancer Research.

[CR45] Thomas ML, de Antueno R, Coyle KM, Sultan M, Cruickshank BM, Giacomantonio MA, Giacomantonio CA, Duncan R, Marcato P (2016). Citral reduces breast tumor growth by inhibiting the cancer stem cell marker ALDH1A3. Molecular Oncology.

[CR46] Tsavaris N, Kopterides P, Kosmas C, Efthymiou A, Skopelitis H, Dimitrakopoulos A, Pagouni E, Pikazis D, Zis PV, Koufos C (2008). Gabapentin monotherapy for the treatment of chemotherapy-induced neuropathic pain: A pilot study. Pain Medicine.

[CR47] Vidovic D, Huynh TT, Konda P, Dean C, Cruickshank BM, Sultan M, Coyle KM, Gujar S, Marcato P (2020). ALDH1A3-regulated long non-coding RNA NRAD1 is a potential novel target for triple-negative breast tumors and cancer stem cells. Cell Death and Differentiation.

[CR48] Warrack BM, Hnatyshyn S, Ott KH, Reily MD, Sanders M, Zhang H, Drexler DM (2009). Normalization strategies for metabonomic analysis of urine samples. Journal of Chromatography b: Analytical Technologies in the Biomedical and Life Sciences.

[CR49] Xiao W, Boroujerdi A, Bennett GJ, Luo ZD (2007). Chemotherapy-evoked painful peripheral neuropathy: Analgesic effects of gabapentin and effects on expression of the alpha-2-delta type-1 calcium channel subunit. Neuroscience.

[CR50] Yang ZL, Yang L, Zou Q, Yuan Y, Li J, Liang L, Zeng G, Chen S (2013). Positive ALDH1A3 and negative GPX3 expressions are biomarkers for poor prognosis of gallbladder cancer. Disease Markers.

[CR51] Yoneda T, Williams PJ, Hiraga T, Niewolna M, Nishimura R (2001). A bone-seeking clone exhibits different biological properties from the MDA-MB-231 parental human breast cancer cells and a brain-seeking clone in vivo and in vitro. Journal of Bone and Mineral Research.

[CR52] Young SZ, Bordey A (2009). GABA’s control of stem and cancer cell proliferation in adult neural and peripheral niches. Physiology (bethesda, Md).

[CR53] Yuan M, Breitkopf SB, Yang X, Asara JM (2012). A positive/negative ion-switching, targeted mass spectrometry-based metabolomics platform for bodily fluids, cells, and fresh and fixed tissue. Nature Protocols.

[CR54] Zhang W, Liu Y, Hu H, Huang H, Bao Z, Yang P, Wang Y, You G, Yan W, Jiang T, Wang J, Zhang W (2015). ALDH1A3: A marker of mesenchymal phenotype in gliomas associated with cell invasion. PLoS ONE.

