# Supplementary material for: Metabolite profiling reveals a connection between aldehyde dehydrogenase 1A3 and GABA metabolism in breast cancer metastasis
Source: Metabolomics. 2022 Jan 6;18(1):9. doi: 10.1007/s11306-021-01864-6 (PMC8739322; doi:10.1007/s11306-021-01864-6)
Supplement: Supplementary file 4 — Supplementary file4 (PDF 125 kb) [file 11306_2021_1864_MOESM4_ESM.pdf]

Supplemental Figures

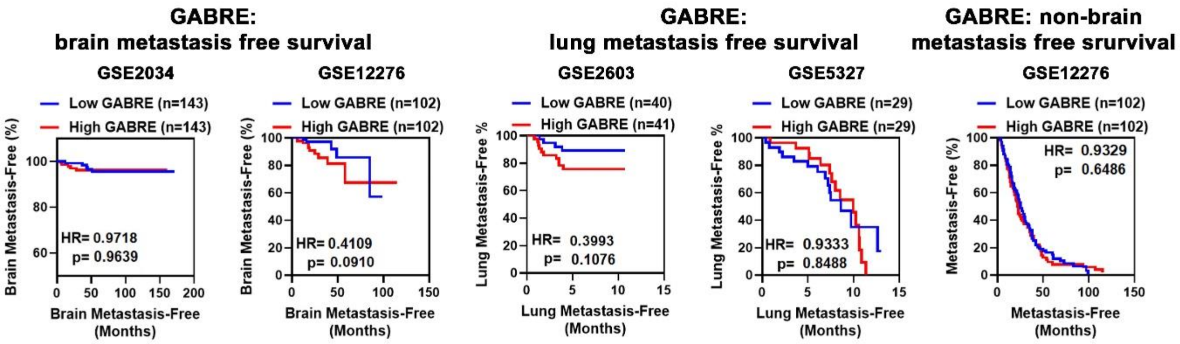

Figure S1. GABRE expression does not correlate with mestastasis development in breast cancer patients. High and low expression of GABRE was categorized based on median expression for Kaplan-Meier metastasis-free survival analysis to the brain, lungs or to sites other than the brain for GSE2034 (Affymetrix Human Genome U133A Array log2 expression from primary tumours of 286 lymph node negative breast cancer patients); GSE12276 (Affymetrix Human Genome U133 Plus 2.0 Array log2 expression of 204 primary breast tumours from patients all of whom experienced local or distant relapse), GSE2603 (Affymetrix Human Genome U133A Array log2 expression from primary tumours of 81 breast cancer patients), and GSE5327 (Affymetrix Human Genome U133A Array log2 expression from 48 primary ER- breast tumours). HR = hazard ratio, p value calculated by log-rank test.
